# Retraction: A High-Throughput Screening-Compatible Strategy for the Identification of Inositol Pyrophosphate Kinase Inhibitors

**DOI:** 10.1371/journal.pone.0180272

**Published:** 2017-06-21

**Authors:** Brandi M. Baughman, Huanchen Wang, Yi An, Dmitri Kireev, Michael A. Stashko, Henning J. Jessen, Kenneth H. Pearce, Stephen V. Frye, Stephen B. Shears

The authors of the above paper retract this article due to concerns about the integrity of the data and the validity of the conclusions. The first author, Brandi M. Baughman, has admitted to the co-authors and the Office of Research Integrity at NIH that she falsified and/or fabricated data and text concerning Figs 2, 3, 4, 5, 6, 8, S1, S2, S3, S4, and S5, for which she takes sole responsibility.

Subsequent to the paper being published, further experiments by co-authors Wang, Stashko, and Pearce have verified that a major conclusion of this article is invalid: UNC10112646, UNC10225354, and UNC10225498 are not inhibitors of PPIP5K, contrary to the claims in the published paper.

In light of these concerns, all of the authors have agreed to retract this article.
